# Repeated Sprint Ability and Muscular Responses According to the Age Category in Elite Youth Soccer Players

**DOI:** 10.3389/fphys.2019.00175

**Published:** 2019-03-06

**Authors:** Javier Sánchez-Sánchez, Jorge García-Unanue, Enrique Hernando, Jorge López-Fernández, Enrique Colino, Manuel León-Jiménez, Leonor Gallardo

**Affiliations:** ^1^School of Sport Sciences, Universidad Europea de Madrid, Villaviciosa de Odón, Spain; ^2^IGOID Research Group, University of Castilla-La Mancha, Toledo, Spain; ^3^Centre for Innovative Research Across the Life Course (CIRAL), Coventry University, West Midlands, United Kingdom

**Keywords:** RSA, soccer, age, tensiomyography, fatigue, muscle

## Abstract

The aim of this study was to analyse the influence of age category on the performance and muscle response after a Repeated Sprint Ability (RSA) test in elite youth soccer players. 62 soccer players from three different age categories (Under 14 [*n* = 21], Under 16 [*n* = 20], and Under 18 [*n* = 21]) were selected to participate in this study. Players completed an RSA test (7 × 30 m) with a 20-s recovery between sprints. The muscular response to an electrical stimulus before and after the test of both the biceps femoris (BF) and the rectus femoris (RF) were evaluated using tensiomyography. A two-way ANOVA was used to analyse the differences in RSA parameters in each of the four distance-intervals (0–5; 5–25; 25–30; 0–30 m) between sprint and age category. The U14 age category (5.30 ± 0.30 s) showed higher mean sprint times than U16 (4.62 ± 0.20 s) and U18 (4.46 ± 0.17 s) throughout the entire test (*p* < 0.01). U16 players revealed a worse best sprints time (RSA_BEST_) than U18 players (+0.12 s, CI95%: to 0.01 to 0.24; ES: 1.09, *p* = 0.03). The muscular contractile properties were similar in the three age categories analyzed (*p* > 0.05), although the delay time (Td) of the muscle was significantly lower after the RSA test in U16 players (−1.53 ms, CI95%: −2.607 to −0.452; ES: 0.38) and U18 players (−1.11 ms, CI95%: −2.10 to −0.12; ES: 0.22). In conclusion, this study revealed an increase in physical performance and muscle response variability after a repeated sprint ability test in the U16's and over. The fatigue induced by the RSA test did not show differences depending on the age of the players, although muscle mechanical properties were altered after the RSA test in U16 and U18 soccer players. Physical performance and muscle response can be complementary variables in managing fatigue according to the age category in soccer players.

## Introduction

During soccer practice, several capacities such as cardiovascular endurance and the ability to perform repeated sprint actions have shown to be decisive for players' performance (Rampinini et al., 2007). For youth players, maturity status is a key variable in their physical outcomes, especially in relation to their physical capacities and match running performances (Buchheit and Mendez-Villanueva, 2014). Previous research has pointed out that the repeated sprint ability (RSA) is related to physiological parameters such as maximal oxygen uptake or muscle phosphocreatine degradation/resynthesis among others (Spencer et al., 2005). Recent research has reported percentile values of repeated sprint performance of young soccer player classified by maturity status (Selmi et al., 2018).

According to Falk and Dotan (2006), adults have a slower recovery from sprint exercise than children. This could be attributed to the fact that children's metabolism is less glycolysis dependent during prolonged recurrent sprints (Hebestreit et al., 1993). The sprint time decreases from the ages of 12–18 years, but at the beginning of adolescence the growth effect has more influence than at the end of this period (Mendez-Villanueva et al., 2011; Malý et al., 2015; Nikolaidis et al., 2016). The biological maturation varies substantially in duration and timing among individuals (Baxter-Jones et al., 2005). Recent studies have focused on the anthropometric and physical profiles (Perroni et al., 2018a; Selmi et al., 2018) but it is surprising that limited information exists on RSA in elite youth soccer players (Mujika et al., 2009), specifically about muscle responses of the players.

Fiber composition is the main factor that decides the speed of muscle contractions (Cormie et al., 2011). A skeletal muscle biopsy is the most accurate test used to collect information on the fiber type composition. Thus, to study the changes on the types of muscle fibers during a child growth it would be necessary to execute repeated biopsies from different muscles over the years. However, due to the great muscle damage that it induces and the ethical issues that might represent to do that in children, alternative methods such as tensiomyography (TMG) could be used to solve this problem.

Tensiomyography is a non-invasive method to assess the contractile properties of skeletal muscles (Valencic et al., 2001; Šimunič, 2012). The TMG applies a stimulation of electrical contraction in basal conditions on the skin, producing a displacement due to an involuntary muscular contraction that is measured with a digital transducer that is in direct contact with the muscular belly (Valencic et al., 2001). This tool has demonstrated a high short-term reliability and sensibility for detecting changes in muscular responses (KriŽaj et al., 2008; Rey et al., 2012); with a greater reliability in a fatigued state than in rested (Ditroilo et al., 2013). Moreover, this method has been demonstrated to predict 87% of the difference in the proportion of type I myosin heavy chain in the vastus lateralis (VL), and has provided reliable information on the fiber composition in children of maturation age, showing a transformation from slow to fast fibers between 6 and 10 years old (Simunic et al., 2017). Therefore, the TMG is a useful tool to compare muscle contractile properties according to age, and it could be used to fill the knowledge gap in the muscle response of young soccer players.

Among the tasks of physical trainers, is the quantification of training loads and the design of tasks according to the age, ensuring the highest efficiency of athletes' training, and optimizing the performance. However, there is a lack of knowledge on muscle performance and muscle contractile properties in young soccer players (Rumpf et al., 2011) after RSA test. Therefore, this study aims to analyse the influence of age category on performance and muscle response after an RSA test in young elite soccer players. The hypothesis proposed in this study was that older soccer players would show lower total times in the RSA test. On the other hand, younger soccer players have a greater ability to recover between sprints and a better contractile response of the muscles of the lower body after a high intensity effort with incomplete recovery.

## Materials and Methods

### Participants

A total of 62 elite youth soccer players (14.63 ± 2.00 years; 167.3 ± 10.5 cm; 58.75 ± 12.52 kg) from three age categories (U14 [*n* = 21], U16 [*n* = 20], and U18 [*n* = 21]) participated in this study. Participants were recruited from an elite soccer academy, this academy signed an agreement to allow the researchers to carry out this study. A parent or guardian of each participant signed an informed consent allowing them to participate in the study. Test procedures and the possible risks of the procedures were explained in detail in the written consent. Moreover, all volunteers who presented at the medical examination, which was required to play soccer, players did not report any cardiopulmonary pathology or other diseases and did not take any medication during the study. The study protocol was approved by the Local Ethics Committee (Hospital of Toledo; CEIC61) and was conducted in accordance with the Code of Ethics of the World Medical Association (Declaration of Helsinki).

### Experimental Design

The data collection process took place during the third month of the regular season. The research team arranged with the soccer teams 2 days within the given period to allow the players to perform the proposed test. Participants agreed not to perform any exhausting activity 48 h before the trials. Prior to the beginning of the study, players completed a familiarization session with both the tests and the equipment included in the study protocol. The main part of the test consisted of a Repeated Sprint Ability Test (RSAt) whose effect was assessed through tensiomyographic measurements obtained right before and after the test. Testing was performed on the artificial turf pitches where participants regularly trained, in dry conditions (temperature: 21 ± 2°C; relative humidity: 20 ± 5%; wind speed: 0.0–0.5 m/s). All tests were carried out within each team's training schedule to avoid the results being affected by circadian rhythm.

### Experimental Protocol

Before completing the RSAt, participants carried out a standardized warm-up protocol consisting of 5 min of continuous running, 5 min of articulation mobility and three 30-meter sprints at increasing intensity. The warm-up concluded with two 30-meter sprints at maximum speed separated by 4 min of active recovery (participants had to walk during the resting time). The best time in these two sprints was used later as a control measure to guarantee players performed the RSAt at maximum intensity. If the time of the first sprint of the RSAt was higher (>5%) than the best individual sprint performed before, the RSAt was not considered valid and the player had to repeat the test after 5 min of recovery.

#### Repeated Sprint Ability Test (RSAt)

The RSAt included seven repetitive sprints of 30 min, with 20 s of active recovery between sprints. Four pairs of photoelectric cells (Witty, Microgate, Bolzano, Italy) were placed at 0, 5, 25, and 30 meters, they were used to assess the performance in this test. Time measurements in four distance-intervals (0–5; 5–25; 25–30; 0–30 meters) were obtained and compared between the groups (U14, U16, and U18). Also, the RSAt sprint time (RSA Time), the RSAt best sprint time (Best Sprint x 7 [RSA_BEST_]), the RSAt mean time (RSA_MEAN_), the RSAt percent decrement (RSA_DEC_) and the difference between the first and last sprint (RSA_CHANGE_) were estimated and compared between the groups.

#### Tensiomyography (TMG)

Muscle response of both the rectus femoris (RF) and biceps femoris (BF) of the players' dominant leg was assessed through tensiomyography (TMG-100 System electrostimulator, TMG-BMC d.o.o., Ljubljana, Slovenia) before and after the RSAt. This mechanism provides information on the muscles' contractile properties as a response to an induced contraction caused by an external electric stimulus. The electric stimulus was induced through two self-adhesive electrodes (TMG electrodes, Ljubljana, Slovenia) and the muscle response was measured with a digital Dc-Dc transducer Trans-Tek® (GK 40, Ljubliana, Slovenia) placed perpendicular to the muscle belly and equidistant from the self-adhesive electrodes at a distance of 50–60 mm. The positions of the sensor and the electrodes were marked with a permanent marker to ensure that measurements before and after the RSAt were performed at the same point. The electric stimulus was 1 ms and they started with a stimulus of 20 mAp which was increased by 10 mAp each time until it reached 110 mAp. A 15 s rest was left between measurements to minimize the effects of potentiation and fatigue. The variables assessed in this study were the maximum radial displacement of the muscle belly (Dm), contraction time (Tc), and delay time (Td). KriŽaj et al. (2008) previously reported a low error level (0.5 to 2.0 %) and a high reproductivity (ICC: 0.85–0.98) for these three parameters (Dm: 0.98; Tc: 0.97; Td: 0.94). No participants reported uncomfortableness during this test. The RF was assessed with participants in supine position with a 120° knee flexion. The BF was evaluated with participants in prone position and the knee flexed 5° (Šimunič, 2012). All measurements were carried out by the same expert technician.

#### Statistical Analysis

Data are presented as mean ± SD. The SPSS V21.0. software (SPSS Inc, Chicago, IL, USA) was used for data analysis and the level of significance was established at *p* < 0.05. The statistical analysis was divided into four parts. Firstly, a two-way ANOVA was used to analyse the differences in RSA sprint times at each of the four distance-intervals (0–5; 5–25; 25–30; 0–30 m) between the sprint (repeated measure: sprint 1 to 7) and the group (independent measure: U14, U16, and U18). Secondly, the differences between the groups (U14, U16, and U18) in the rest of RSA variables (RSA_MEAN_, RSA_BEST_, RSA_DEC_, and RSA_CHANGE_) was evaluated by means of a one-way ANOVA. Thirdly, a two-way ANOVA was used to analyse the differences in the tensiomyography variables both in BF and RF when taking into account the moment (repeated measure: pre-post) and the group (independent measure: U14, U16, and U18). Bonferroni *post-hoc* analysis was used in all pairwise comparisons in the three previous ANOVA tests. The confidence interval of the differences (CI of 95%) was calculated to identify the magnitude of changes and the effect size (ES; Cohen's d). The ES was evaluated using the following criteria: 0–0.2 = trivial, 0.2–0.5 = small, 0.5–0.8 = moderate and >0.8 = large (Cohen, 1992). Finally, a product-moment correlation (Pearson's r) was established between the results of the RSA test and the values of the muscle response of the dominant leg of the soccer players. Correlations were evaluated following these criteria: 0–0.1 = trivial, 0.1–0.3 = small, 0.3–0.5 = medium, 0.5–0.7 = large, 0.7–0.9 = very large, and 0.9–1.0 = nearly perfect (Hopkins et al., 2009).

## Results

**Figure 1:**
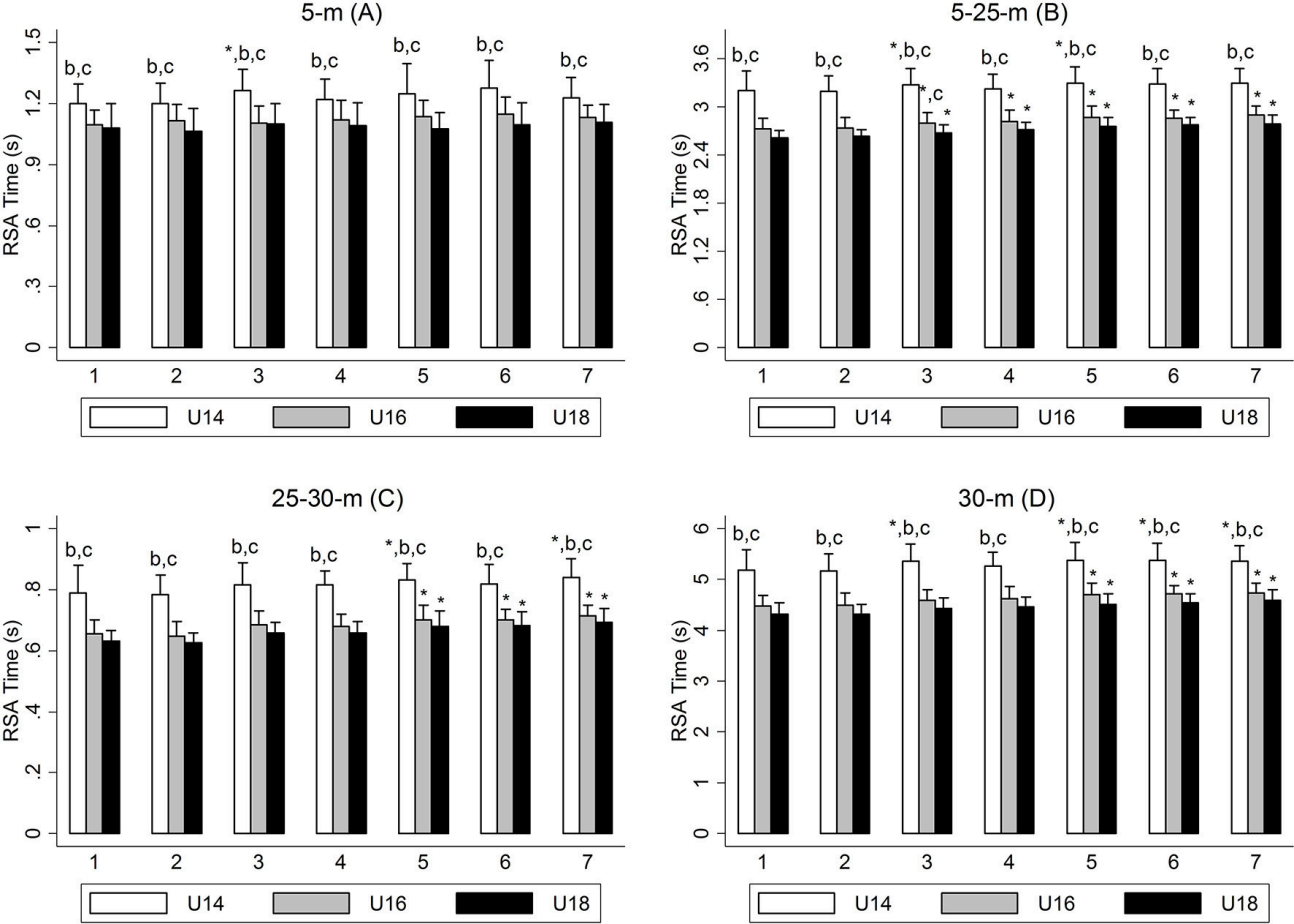
5 **(A)**, 5–25 **(B)**, 25–30 **(C)**, and 30-m **(D)** time and performance deterioration profile for the RSA test (7 × 30m) in U14 (*n* = 21), U16 (*n* = 20), and U18 (*n* = 21) soccer players. *p < 0.05 significantly different from the 1st sprint for 5–25- and 30-m times; ^b^*p* < 0.05 significantly different from U16; ^c^*p* < 0.05 significantly different from U18. Data are presented as mean and SD.

The RSA test results showed better sprint times in the U16 and U18 players in comparison with the U14 players in all the analyzed distance intervals (Figure 1, *p* < 0.05), although the size of the difference was higher between the U14 and U18 players (from 1.11 to 4.08). On the other hand, the U16 players revealed a worse RSA_BEST_ than the U18 players (+0.12 s, CI95%: to 0.01–0.24; ES: 1.09, *p* = 0.03; Table 1). The performance decrement according to the first sprint in the RSA test (30 m) was marked from the fifth sprint in the age categories analyzed (*p* < 0.05). However, RSA_CHANGE_ and RSA_DEC_ were similar between categories (*p* > 0.05).

**Table 1:** Performance (RSA_MEAN_; RSA_BEST_) and fatigue (RSA_DEC_; RSA_CHANGE_) in a Repeated Sprint test (7 × 30 m) in U14, U16, and U18 soccer players.

	**U14 (a)**	**U16 (b)**	**U18 (c)**
RSA_MEAN_ (s)	5.30 ± 0.30^b, c^	4.62 ± 0.20	4.46 ± 0.17
RSA_BEST_ (s)	35.54 ± 2.11^b, c^	31.04 ± 1.44^c^	29.80 ± 1.01
RSA_DEC_ (%)	4.45 ± 1.79	4.21 ± 1.78	4.67 ± 1.97
RSA_CHANGE_ (%)	9.42 ± 5.18	7.77 ± 2.99	8.80 ± 3.19

b *p < 0.05 significantly different from U16; ^c^p < 0.05 significantly different from U18. Data are presented as mean and SD (n = 62)*.

The tensiomyographic variables were similar among the soccer players of the three categories analyzed (*p* > 0.05, Table 2). The results after the RSA test in the RF revealed a significant reduction in the Td in U16 players (−1.53 ms, CI95%: −2.607 to −0.452; ES: 0.38, *p* = 0.006) and U18 players (−1.11 ms, CI95%: −2.10 to −0.12; ES: 0.22, *p* = 0.03), as well as a lower Dm in U16 players after the test (−1.82 mm, CI95%: −2.89 to −0.76; ES: 0.78, *p* = 0.001).

**Table 2:** Results of the tensiomyography before and after the RSA test (7 × 30 m) for different categories (U14, U16, U18) in soccer players.

		**U14**		**U16**		**U18**	
		**Pre**	**Post**	**Pre**	**Post**	**Pre**	**Post**
**BF**							
	Td (ms)	26.34 ± 2.84	24.91 ± 3.66	25.96±.76	26.04 ± 3.57	26.04 ± 3.77	24.92 ± 3.43
	Tc (ms)	38.44 ± 17.66	35.20 ± 15.84	35.21 ± 13.97	33.48 ± 11.03	38.44 ± 14.96	35.30 ± 14.53
	Dm (mm)	5.88 ± 2.59	5.38 ± 2.66	7.92 ± 4.83	6.85 ± 2.96	6.82 ± 2.66	6.24 ± 2.25
**RF**							
	Td (ms)	30.19 ± 3.28	29.83 ± 3.19	28.17 ± 3.63	26.64 ± 4.44^*^	28.93 ± 5.10	27.82 ± 5.06^*^
	Tc (ms)	42.99 ± 7.53	46.64 ± 7.94	40.57 ± 9.92	40.83 ± 9.47	42.49 ± 10.20	44.04 ± 10.34
	Dm (mm)	8.85 ± 2.78	8.12 ± 2.27	10.61 ± 2.28	8.79 ± 2.58^*^	9.86 ± 2.38	9.97 ± 2.88

* *Differences between the pre and post tensiomyographic variables in U14 (n = 21), U16 (n = 20), and U18 (n = 21) soccer players (p < 0.05)*.

Finally, the correlation analysis between the tensiomyographic variables and the performance in the RSA test revealed a significant relationship between the fatigue variables of the test (RSA_DEC_ and RSA_CHANGE_) and the variation of the Dm in the RF of the sample analyzed (*r* = −0.41 and *r* = −0.34, *p* < 0.05, respectively, Table 3). The inclusion of the tensiomyographic values prior to the RSA test and its link with the performance in this test did not show any significant relationship (*p* > 0.05).

**Table 3:** Correlation coefficients between performance (RSA_TT_, RSA_BEST_, RSA_MEAN_) and fatigue (RSA_DEC_, RSA_CHANGE_) variables derived from the RSA test and the variation coefficients of the TMG parameters for the RF and BF.

	**RSA_TT_**	**RSA_BEST_**	**RSA_MEAN_**	**RSA_DEC_**	**RSA_CHANGE_**
**BF**					
Td_CHANGE_	0.06	0.05	0.06	0.04	0.08
Tc_CHANGE_	−0.04	−0.07	−0.04	0.10	0.11
Dm_CHANGE_	−0.07	−0.09	−0.07	0.09	0.12
**RF**					
Td_CHANGE_	−0.17	−0.17	−0.17	0.01	−0.12
Tc_CHANGE_	0.01	0.02	0.01	−0.07	−0.18
Dm_CHANGE_	0.12	0.19	0.12	−0.410^**^	−0.340^*^

* *p < 0.05*;

** *p < 0.01; Dm, maximum radial displacement of muscle belly; Tc, contraction time, Td, delay time, Ts, sustained contraction time; Tr, half-relaxation time; RF, rectus femoris; BF, biceps femoris (n = 62)*.

## Discussion

The purpose of this study was to analyse the influence of the age category on the 7 × 30 meter RSA test performance in elite youth soccer players and the effect of this test on the mechanical behavior of the biceps femoris and rectus femoris of these players. The main results of this research were that U14 players presented a higher sprint time than the other two age categories, but no differences were found between the U16 and U18 players. However, the performance decrement during the RSA was not age-related. Regarding muscular responses to the electric stimulus, no differences were found among age category, but U16 and U18 players evidenced lower Td responses, Dm responses, and Td responses, respectively, on the rectus femoris after the RSA.

When analyzing the findings of previous studies on the influence of age in sprint performance, we can conclude that the sprint time improves from the ages of 12 to 18 years old, but the age effect is larger at the beginning of adolescence (Mendez-Villanueva et al., 2011; Nikolaidis et al., 2016). Indeed, Mendez-Villanueva et al. (2011) found that the 15-year-old group ran 10 meters faster than the 13-year-old group (−0.13 s), but slower (+0.07 s) than the 17-year-old group. These assumptions are also evidenced in our research since U14 players run the 7 × 30 meter RSA test slower than U16 and U18 players; whereas U16 players presented a higher sprint time than U18 players for the RSA_BEST_ (+0.122 s). Therefore, it seems that the biggest improvement in linear sprint performance happens at the end of the U14 player period. In contrast, the better RSA_BEST_ in U18 players when compared to U16 players may be explained by the lower power and muscle mass of U16 players (Zafeiridis et al., 2005); suggesting that after the U16 age group, training is the most responsible for improving performance instead of maturation (Di Mascio et al., 2017). These findings are in line with Stratton et al. (2004) who showed that the peak height velocity in soccer players occurs from 13.8 to 14.2 years old. Nonetheless, sprint performance may be influenced by internal factors, such as biological maturity age or running technique (Buchheit and Mendez-Villanueva, 2014; Haugen et al., 2014).

The strong evidence that older adolescents run faster than younger ones (Mendez-Villanueva et al., 2011; Nikolaidis et al., 2016) may suggest that older teenagers also run faster during the RSA. Our findings partially support this hypothesis as U14 players achieved a lower sprint performance than the other two age groups (U16 and U18). However, U16 players did not evidence lower RSA performance than U18 players. These results are in line with those of Philippaerts et al. (2006) who evidenced a greater improvement in speed capacity in young players' right before and after their peak height velocity (13.8 ± 0.8 years old in male adolescents) whereas the anaerobic capacity of the soccer players showed a spurt at the time of peak height velocity. Moreover, Malina et al. (2004) reported an improvement in strength, power, and speed at this age. Therefore, these outcomes suggest that the speed training should be more specific from the U16 age category onwards. Whereas, the use of RSA protocols for talent identification should be applied once players surpass the peak height velocity period (Perroni et al., 2018b; Selmi et al., 2018). It is important to consider that the characteristics of each repeated-sprint ability test (recovery duration, number of sprints, distance, etc.) can influence the mean sprint time (Spencer et al., 2005). For instance, those protocols that include several sets of repeated sprints showed higher differences between the performance of the U16 and U18 players (Selmi et al., 2018). When comparing the RSA_MEAN_ performance of this study with previous studies, we found that U14 and U18 soccer players selected for this study ran slower (+0.26 and +0.07 s, respectively) and presented a higher standard deviation (+0.02 and +0.05, respectively) than those chosen by Mendez-Villanueva et al. (2011). Whereas, U16 soccer players participating in the present study did not present a lower RSA_MEAN_, but a greater standard deviation (+0.03). Therefore, it is likely that our findings were affected by the lower repeated-sprint ability of the U14 and U18 groups included in our study. Perroni et al. (2018b) found a great variability in physical parameters like speed time even in young soccer players from the same category. Therefore, training loads should be in line with the individual maturity status of these players.

Regarding the capability of young soccer players to deal with incomplete recovery, previous studies have revealed that the ability to repeat short-term maximal efforts (linear and non-linear sprints) declines as age increases (Falk and Dotan, 2006; Ratel et al., 2006). In the present study, RSA_DEC_ and RSA_CHANGE_ variables inform about the ability of players to sustain the sprint performance for a period of time (Glaister et al., 2008; Buchheit, 2010). Therefore, the lack of differences in these variables suggest that the high-intensity profile in U14 soccer players is slower than in U16 and U18 players, but they do not have different capacities to face incomplete recovery than the older groups. These findings coincide with those from Mujika et al. (2009) who found that repeated-sprint ability was not affected by age in elite soccer players aged 11–18 years old. Our findings reaffirm the findings of Bishop and Edge (2006), they revealed that repeated-sprint ability is positively correlated to aerobic fitness and negatively related with the anaerobic contribution of the first sprint. Thus, our expectation of higher RSA_DEC_ and higher RSA_CHANGE_ in U14 players than in U18 players was rejected. These findings suggest that the three age groups assessed in this study can face exercises that required the repetition of high intensity actions or sprints in a short period of time. However, coaches should control the fitness condition and body mass of their players, as the ability of young soccer players to deal with repeated sprinting actions are correlated to these variables (Perroni et al., 2018b).

On the other hand, the contractile properties of the muscles of the lower limb are related with the ability of these muscles to maintain or even increase performance in subsequent efforts and mitigate the injury risk especially in fatigued conditions (Rey et al., 2012; Malone et al., 2017; Simunic et al., 2017). In this study, the TMG has been used to assess the contractile responses of the rectus femoris and biceps femoris before and after a 7 × 30 meter RSA, as this tool has demonstrated a high short-term reliability and sensibility for detecting changes in muscular responses (KriŽaj et al., 2008; Rey et al., 2012); with a greater reliability in a fatigued state than in rested (Ditroilo et al., 2013). However, Wiewelhove et al. (2017) doubted that the sensibility of the TMG for detecting acute muscular fatigue; whereas the reliability of the measurement can be affected by the position of the electrodes and the digital displacement transducer, the research design or the recovery time between stimulus, among others (Rey et al., 2012). Therefore, care must be taken when considering these findings.

The lack of differences in basal values of Dm, Td, and Tc showed that the contractile properties of both biceps femoris and rectus femoris is not age-related in teenage soccer players (Simunic et al., 2017). When assessing the basal outcomes of this study we found that teenagers have a low number of type II fibers (Tc higher than > 30 ms; Dahmane et al., 2001; KriŽaj et al., 2008). These findings go against the outcomes from adult soccer players who were shown to have a high number of fast fibers (Tc usually lower than < 30 ms; Rey et al., 2012; Alentorn-Geli et al., 2015; García-García et al., 2017). On the other hand, teenagers have a low Dm value, which suggests that they have a high muscle tone (Dahmane et al., 2005), although these findings may be affected by factors such as fatigue or study design.

In TMG, fatigue is displayed by changes in electric muscle activity; the reduction in the capacity to sustain a determined level of strength during the contraction or the incapacity to reach an initial strength level in repeated contractions (Rodríguez-Matoso et al., 2010). In the present study, no differences before and after the RSA were found in U14 players; but in the rectus femoris after the RSA, U16 players showed lower values of Td and Dm, U18 players showed a lower Td. These findings may provide evidence that younger teenagers have a higher ability to recover from high-intensity exercise (Di Mascio et al., 2017); probably because the phosphocreatine (PCr) resynthesis is faster in children (Hebestreit et al., 1993; Taylor et al., 1997). However, further studies are required as our correlation analyses showed that the greater performance decrement of youth soccer players during the RSA is related to a lower variation in Dm values of the rectus femoris (moderate negative correlation [0.3–0.5] between players' fatigue [RSA_DEC_ and RSA_CHANGE_] and the variation of Dm [Dm_CHANGE_] in the rectus femoris.

From a practical point of view, the results presented in this study suggest that repeated sprints in young soccer players should provide higher muscular adaptations from U16 category onwards, so training RSA actions should increase in importance in these categories (Mujika et al., 2009). However, training RSA actions in U14 players should not be avoided as they are able to cope with the demands of RSA actions like U16 and U18 players. Finally, despite no differences in performance deterioration are presented among the three age categories assessed in this study, it is important to highlight that U16 and U18 show higher muscular responses to RSA test than U14. Coaches of U16 and U18 teams need to control the volume of training sessions that include repeated sprints to reduce the risk of muscular injuries.

Finally, it is necessary to consider that this study only assessed the acute response of players' rectus femoris and biceps femoris. Therefore, further analysis 24–48 h after the RSA would provide further information on how the RSA load varies according to the age and if the recovery strategies are different in these age groups. Moreover, the findings of this study belong to the third term of competition, so cautiousness must be taken when comparing them with other competition periods.

## Conclusion

This study revealed an increase in performance in repeated sprints from U16 players, at which time the ability to perform this type of action is equalized. In this sense, the deterioration of performance in the RSA test was homogeneous in the three age groups analyzed. Despite this, the contractile properties of the lower limbs in U14 soccer players remained unchanged after the RSA test, with values similar to those of the older players. Finally, the maximum radial displacement of the muscle belly showed a significant relationship with the decrease in the capacity to perform high intensity efforts with incomplete recovery in soccer players, muscle mechanical properties were altered after the RSA test in U16 and U18 soccer players. Physical performance and muscle response can be complementary variables to managing the fatigue according to the age category in soccer players. These data shows the need to adjust the training load according to the age of the soccer players, especially in the repeated-sprint ability which is considered as a relevant parameter for obtaining success in this sport.

## Ethics Statement

Parents and children were informed about the research goal and its procedure, as well as its possible risk. Children gave their consent verbally and their parents signed the written informed consent. The study protocols were approved by the ethical committee from the University of Castilla–La Mancha (Toledo, Spain) on 10 May 2015, according to the Helsinki Declaration about ethic principles of medical research in humans. All measurements were taken in the same condition, following the same actuation protocol with each participant. All evaluations were done from September to October 2016.

## Author Contributions

JS-S and LG conceived the presented idea. EC and EH developed the background and performed the calibration of the different devices used in the tests. JG-U verified the methods section. All authors discussed the results and contributed to the final manuscript. JL-F, EC, and ML-J carried out the tests, JS-S wrote the manuscript with support from JL-F and EC. LG helped supervise the project. Both JS-S and JG-U contributed to the final version of the manuscript. ML-J and JG-U contributed to the interpretation of the results and data analysis and they drafted the manuscript and designed the figures and tables. All authors provided critical feedback and helped shape the research, analysis, and manuscript.

### Conflict of Interest Statement

The authors declare that the research was conducted in the absence of any commercial or financial relationships that could be construed as a potential conflict of interest.
